# Modified Das De Procedure in Peroneus Longus Tendon Dislocation: Technical Note

**DOI:** 10.1002/atn2.70180

**Published:** 2026-08-13

**Authors:** I. Wayan Subawa, Febyan Febyan

**Affiliations:** ^1^ Consultant of Foot and Ankle Division, Department of Orthopaedic and Traumatology, Faculty of Medicine Udayana University, Prof. Ngoerah General Hospital Bali Indonesia; ^2^ Department of Orthopaedic and Traumatology, Faculty of Medicine Udayana University, Prof. Ngoerah General Hospital Bali Indonesia

## Abstract

Peroneal tendon dislocation is an uncommon ankle injury that may be misinterpreted as a lateral ankle sprain due to similar clinical characteristics. It frequently leads to delayed treatment and recurrent instability. Although various surgical approaches have been described, including osseous procedures such as groove deepening and soft‐tissue reconstruction, no single technique has been universally accepted as the gold standard for treating peroneal tendon dislocation. This technical note describes a modified Das De procedure for the treatment of recurrent peroneus longus tendon dislocation (Eckert and Oden type A). Preoperative imaging in affected patients may show peroneal tenosynovitis without evident tendon dislocation. However, intraoperative observations validated intact tendons with superior peroneal retinaculum insufficiency. The retinaculum is repaired and fortified, and postoperative provocative testing typically shows stable tendon positioning without redislocation. By preserving anatomical integrity and minimizing osseous manipulation, this modified procedure provides a safe, reproducible, and effective alternative for treating symptomatic peroneal tendon dislocation in young and active patients.

## Graphical abstract

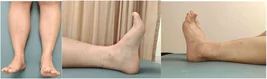

**VIDEO 1:** Step‐by‐step illustration of the modified Das De procedure for stabilization of recurrent peroneus longus tendon dislocation in a left ankle. The patient is positioned supine under general anesthesia. A posterolateral incision is made posterior to the distal fibula to expose the superior peroneal retinaculum and peroneal tendons. The peroneus longus and peroneus brevis tendons are identified, inspected, and reduced into the retromalleolar groove. The superior peroneal retinaculum is refreshed and reconstructed using local fascial tissue to reinforce the retinacular restraint without bone modification. Final intraoperative provocative testing with ankle dorsiflexion and eversion confirms stable positioning of the peroneus longus tendon without redislocation, showing effective restoration of tendon stability. Video content can be viewed at https://doi.org/10.1002/atn2.70180.

Peroneal tendon dislocation (PTD) is an uncommon injury of the lateral ankle, comprising only 0.3% to 0.5% of all ankle traumas.^1^, ^2^ As described in the case presented by Lohrer et al., peroneal tendon dislocation may result from repetitive mechanical irritation, overuse, detachment, or rupture of the retinacular structures, which can be associated with overpronation and friction from footwear.^3^ It typically arises from forceful dorsiflexion of a slightly everted foot, followed by abrupt contraction of the peroneal muscles, frequently observed in sports that necessitate rapid directional changes: soccer, basketball, and gymnastics.^4^, ^5^ The superior peroneal retinaculum (SPR) and fibrocartilaginous ridge serve as stabilizers, inhibiting anterior subluxation of the peroneus longus (PL) and peroneus brevis (PB) tendons across the lateral malleolus. Disruption of these structures can lead to acute dislocation, which, if misdiagnosed or improperly managed, may advance to recurrent post‐traumatic dislocation.^4^

Clinically, peroneal tendon dysfunction (PTD) is frequently misdiagnosed as a lateral ankle sprain because to the similarity in symptoms, including pain, edema, and tenderness in the lateral ankle area. Nonetheless, although ankle sprains generally exhibit tenderness anterior to the malleolus, posterior tenderness is indicative of PTD.^4^ This diagnostic uncertainty results in insufficient recognition and postponements in appropriate therapy. Imaging modalities, particularly ultrasonography and magnetic resonance imaging (MRI), can enhance diagnostic accuracy.^5^, ^6^ Nevertheless, initial cases may sometimes be overlooked, resulting in persistent instability, repeated snapping, or tendon degeneration.^4^

Notwithstanding efforts at conservative treatment via immobilization, the failure rate is significantly elevated, especially among athletes.^4^, ^6^ Consequently, surgical intervention is the primary treatment for symptomatic or recurrent peroneal tendon dysfunction (PTD).^5^ The Das De procedure, a soft tissue procedure originally designed for peroneal tendon instability, has been widely used with several modifications to improve outcomes and reduce complications. In cases involving the PL tendon, improved procedures can provide secure tendon positioning while minimizing iatrogenic injury.^4^ This technical note presents a modified Das De procedure for stabilizing the PL tendon in cases of recurrent dislocation, highlighting its role as an effective alternative to conventional methods.

## SURGICAL TECHNIQUE

**FIGURE 1:**
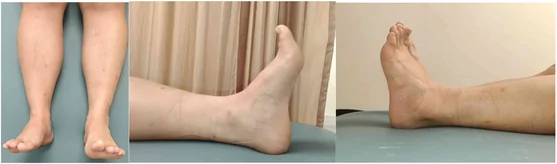
Preoperative clinical appearance of the lateral aspect of the left ankle showing the anatomical region commonly associated with peroneal tendon instability. This area corresponds to the course of the peroneal tendons posterior to the lateral malleolus.

This technique is indicated for patients presenting with chronic lateral ankle pain and recurrent PL tendon instability. The patient is extremely active and consistently participated in sports, necessitating repetitive foot and ankle motions. Symptoms typically occur following traumatic ankle dorsiflexion with inversion or eversion (Figure 1). Conservative management, including analgesics and rehabilitation, may fail to alleviate symptoms in active patients. A provocative test is performed by applying ankle dorsiflexion and eversion, which may reproduce PTD.

**FIGURE 2:**
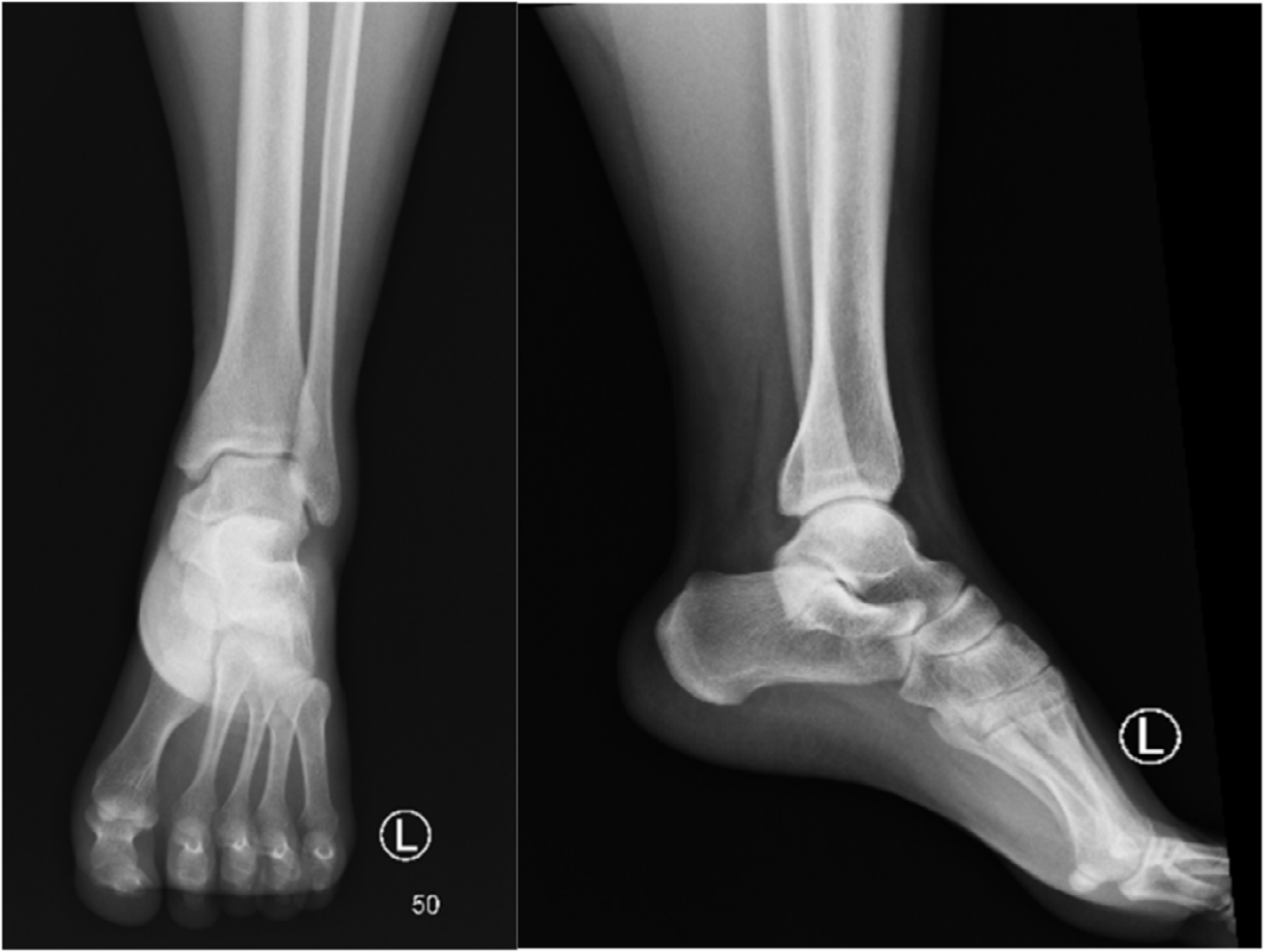
Preoperative plain radiographs of the left ankle in anteroposterior and lateral views showing no osseous abnormalities, fractures, or fibular groove deformities associated with peroneal tendon instability.

**FIGURE 3:**
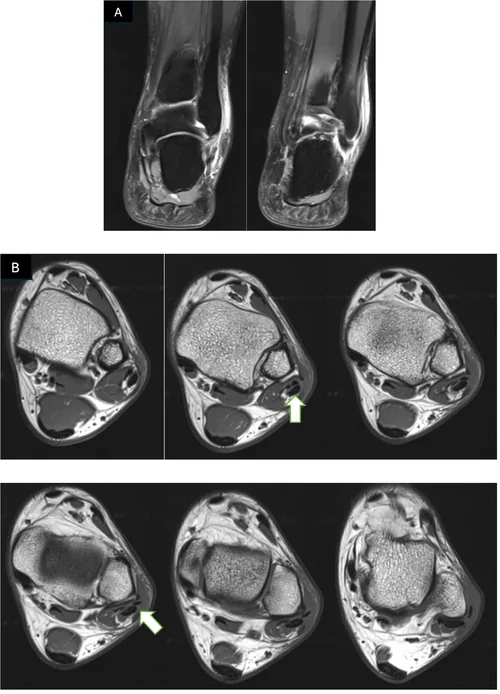
Preoperative magnetic resonance imaging of the left ankle showing (A) coronal and (B) axial views. The images show peroneal tendon tenosynovitis without evident tendon dislocation or subluxation, highlighting the limitation of static imaging in detecting dynamic peroneal instability.

Thereafter, imaging studies are conducted. Plain radiographs are often inconspicuous in patients with peroneal tendon instability (Figure 2). MRI of the left ankle without contrast showed pathological abnormalities, including tenosynovitis of the PL and brevis tendons, along with soft tissue edema surrounding the ankle. No indications of chronic systemic disease or autoimmune condition are detected (Figure 3).

Laboratory examinations indicated results within the usual range. Based on the physical examination, we suspected dislocation of the PL tendon classified as type A according to the Eckert and Oden classification. Consequently, surgical stabilization using a modified Das De procedure is recommended in symptomatic or recurrent cases.

### Patient Positioning and Landmark Incision

**FIGURE 4:**
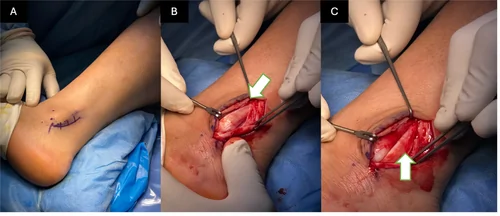
Intraoperative exposure of the left ankle during the modified Das De procedure. (A) A posterolateral skin incision is made posterior to the lateral malleolus. (B) The superior peroneal retinaculum is identified after careful soft‐tissue dissection. (C) The peroneus longus and peroneus brevis tendons are exposed and inspected within the peroneal tendon sheath.

The patient is in a supine posture under general anesthesia, with the left leg draped. A posterolateral skin incision is performed (Figure 4), followed by hemostasis and a deep incision. Fascia and SPR tissue are identified, and the SPR is dissected to reveal the PL and brevis tendons.

### Identified PL and PB Tendons

**FIGURE 5:**
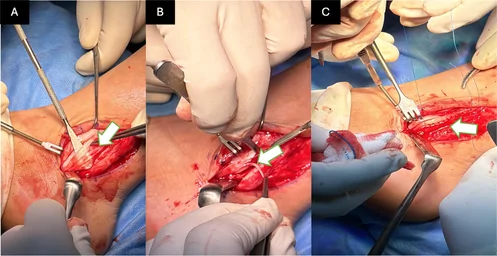
Intraoperative steps of SPR reconstruction in the left ankle. (A) The peroneus longus and peroneus brevis tendons are released and identified. (B) The SPR tissue is refreshed to promote healing. (C) The SPR is sutured after reduction of the peroneus longus tendon into its anatomical position within the retromalleolar groove. (SPR, superior peroneal retinaculum.)

Following a deep incision, the PL and PB tendons are identified, and the SPR tissue is released from the tendons; both PL and PB remained intact (Figure 5A,B).

### Identification and Reconstruction of SPR

**FIGURE 6:**
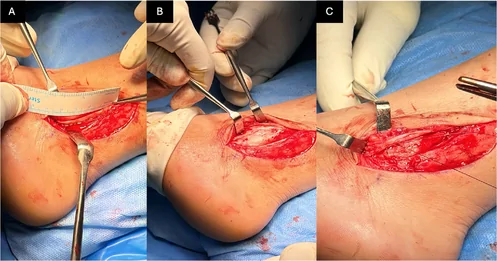
Operative illustration of the left ankle: (A) after suturing all the soft tissue from the SPR and peroneus longus tendon, the measured length was 5 cm. (B) Identification of fascia and SPR layers. (C) Suturing fascia with SPR layer. (SPR, superior peroneal retinaculum.)

The SPR tissue is incised to renew the tissue, then sutured after repositioning the PL, and the SPR tissue is recreated using fascia and a layer of the PL tendon (Figures 5C and 6A‐C).

### Re‐Evaluation and Provocative Test

**FIGURE 7:**
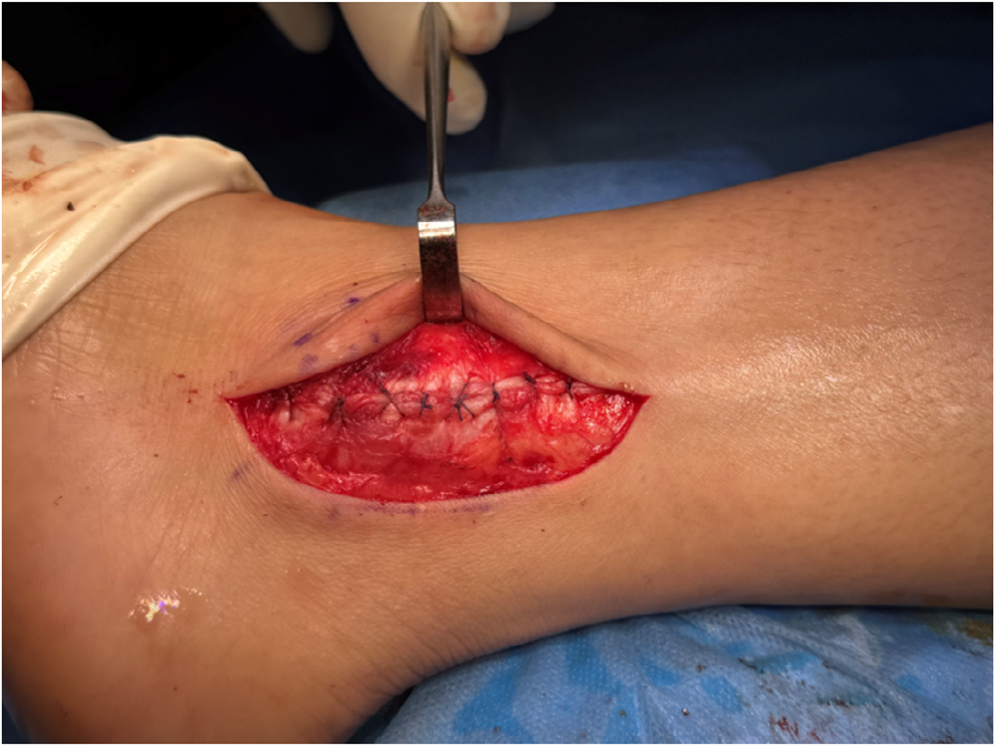
Final intraoperative appearance of the left ankle after completion of superior peroneal retinaculum reconstruction and layered soft‐tissue closure.

Following the rebuilding of the SPR and the repositioning of the PL tendons, we conducted a provocative test and saw during the procedure that the PL tendon remained stable and secured in its anatomical position (Figure 7 and Video 1). Postoperative outcomes generally show improved ankle stability and functional recovery.

## DISCUSSION

PTD is an uncommon yet clinically important etiology of persistent lateral ankle pain and dysfunction, often misidentified as a mere sprain. In cases of recurrent PL tendon dislocation, clinical instability may persist despite inconclusive imaging findings. This underscores the significance of provocative testing and meticulous clinical correlation, as MRI may inadequately reflect dynamic pathology.^7^, ^8^, ^9^

The SPR and the architecture of the retromalleolar groove are essential stabilizers of the peroneal tendons. Groove deepening procedures maintain peroneal tendons and avert dislocation by enhancing the groove on the posterior wall of the fibula that accommodates the peroneal tendon at the ankle level.^4^ Variations in groove morphology concave, flat, or convex have been identified as predisposing factors.^10^, ^11^, ^12^ A recent study revealed that groove morphology varies according to the computed tomography (CT) slice level, with up to 70% of patients exhibiting inconsistent classifications, thereby highlighting the intricacies of anatomical predispositions.^13^ Additionally, the presence of fibrocartilage lining, rather than bone depth, may influence tendon containment.^14^

MRI is effective for detecting concomitant injuries, including SPR tears, peroneal tenosynovitis, and longitudinal tendon splits.^15^, ^16^, ^17^ MRI may reveal tenosynovitis without overt dislocation, emphasizing the importance of clinical correlation.

Nonoperative management, usually including casting or taping, shows suboptimal outcomes in adults, especially sports. Previous studies revealed redislocation rates of up to 60% with taping and 32% with short‐term casting, whereas immobilization for 6 weeks yielded slightly improved results.^4^, ^5^ Consequently, surgical intervention is typically advised for recurrent or symptomatic instances.^18^, ^19^

Operative strategies can be categorized into the following: (1) soft tissue procedures: SPR repair or reattachment; (2) bony procedures: groove deepening, osteotomies, or bone block stabilization; and (3) combined procedures: retinacular repair alongside groove deepening or tendon rerouting.^6^, ^20^ Although groove deepening enhances mechanical stability, apprehensions remain about fibrocartilage injury, fracture susceptibility, and delayed union.^21^ Retinacular repair mitigates these issues and maintains anatomical integrity, yet recurrence has been observed in instances involving shallow grooves.^3^

The Das De procedure is among the initial soft tissue techniques especially developed for recurrent PTD.^22^ It entails strengthening and reattaching the SPR to reestablish stability without modifying the fibular groove.^23^ Altered iterations of this procedure have been formulated to target isolated engagement of the PL or brevis, maintaining tendon mobility and reducing morbidity.^16^, ^24^ Previous studies indicated positive results with modified SPR repair, highlighting diminished complication rates relative to bony interventions.^14^, ^16^ The advantages and disadvantages of the modified Das De procedure are summarized in Table 1.

**TABLE 1:** Advantages and Disadvantages of the Modified Das De Procedure^2^, ^3^

Advantages
• Good clinical outcomes and low recurrence
• Faster return to sport, especially in athletes
• Less complication related to skin incisions/soft tissues
• Preservation of fibrocartilage supports natural tendon gliding
• It allows combination with groove deepening and tendon repair when necessary

Disadvantages
• Potential for recurrence if groove anatomy not adequately addressed
• Associated tendon tears or low‐lying muscle belly may not be fully corrected
• The outcome depends on retinacular tissue quality and healing
• Technical errors in retinaculum tensioning can impair tendon excursion
• Risk of adhesions and stiffness remains if rehabilitation is suboptimal

A modified Das De procedure is executed to stabilize the PL tendon. The approach circumvented osseous manipulation, maintained the fibrocartilaginous surface, and directly targeted the tenosynovitis noted intraoperatively. This method exemplifies the increasing inclination toward less invasive, anatomy‐conserving stabilizing techniques with favorable documented results.^3^, ^23^

The modified Das De procedure provides a more straightforward and less invasive approach to soft tissue stabilization, eliminating the need for bone intervention while ensuring sufficient gliding, hence endorsing its application in specific instances. Pearls and pitfalls are summarized in Table 2.^24^, ^25^

**TABLE 2:** Pearls and Pitfalls^2^, ^8^, ^9^

Pearls
• Preoperative imaging (MRI/US) is key to confirm diagnosis and detect associated lesions
• Preserve fibrocartilage when deepening the fibular groove to maintain tendon gliding
• Always repair or reinforce the SPR for stability
• Early but controlled rehab prevents adhesions and stiffness

Pitfalls
• Inadequate or excessive groove deepening may result in recurrence or instability
• Missed tendon tears or low‐lying muscle belly may lead to persistent symptoms
• Incorrect SPR tensioning may limit excursion or fail to stabilize
• Poor compliance with postop immobilization increases recurrence risk

MRI, magnetic resonance imaging; SPR, superior peroneal retinaculum; US, ultrasonography.

In conclusion, the modified Das De method showed efficacy in this instance, mitigating instability while circumventing osseous problems. It is especially appropriate for young, active patients when prompt mobilization and less morbidity are advantageous. Due to the diverse anatomy of the retromalleolar groove and varying surgical philosophies, personalized surgical planning is crucial. Prospective, comparative studies are required to validate long‐term recurrence and complication rates among procedures.

## DISCLOSURES

The authors (I.W.S., F.F.) declare that they have no known competing financial interests or personal relationships that could have appeared to influence the work reported in this article.
